# A new SNP-based vision of the genetics of sex determination in European sea bass (*Dicentrarchus labrax*)

**DOI:** 10.1186/s12711-015-0148-y

**Published:** 2015-09-04

**Authors:** Christos Palaiokostas, Michaël Bekaert, John B. Taggart, Karim Gharbi, Brendan J. McAndrew, Béatrice Chatain, David J. Penman, Marc Vandeputte

**Affiliations:** Institute of Aquaculture, School of Natural Sciences, University of Stirling, Stirling, FK9 4LA Scotland UK; The Roslin Institute, Royal (Dick) School of Veterinary Studies, University of Edinburgh, Easter Bush, Midlothian, EH25 9RG Scotland UK; Edinburgh Genomics, Ashworth Laboratories, King’s Buildings, University of Edinburgh, Edinburgh, EH9 3JT Scotland UK; Ifremer, Chemin de Maguelone, F-34250 Palavas-les-Flots, France; INRA, UMR1313 Génétique animale et Biologie intégrative, F-78350 Jouy-en-Josas, France

## Abstract

**Background:**

European sea bass (*Dicentrarchus labrax*) is one of the most important farmed species in Mediterranean aquaculture. The observed sexual growth and maturity dimorphism in favour of females adds value towards deciphering the sex determination system of this species. Current knowledge indicates the existence of a polygenic sex determining determination system that interacts with temperature. This was explored by restriction-site associated DNA (RAD) marker analysis in a test panel of 175 offspring that originated from a factorial cross between two dams and four sires from a single full-sib family.

**Results:**

The first high-density single nucleotide polymorphism (SNP) based linkage map for sea bass was constructed, consisting of 6706 SNPs on 24 linkage groups. Indications for putative sex-determining QTL (quantitative trait loci) that were significant at the genome-wide threshold were detected on linkage groups 6, 11 and 18 to 21, although a genome-wide association study (GWAS) did not identify individual significant SNPs at a genome-wide threshold. A preliminary genomic prediction approach that tested the efficiency of SNP-based selection for female sea bass showed a slight advantage compared to traditional pedigree-based selection. However, when the same models were tested on the same animals for selection for greater length, a clear advantage of the SNP-based selection was observed.

**Conclusions:**

Overall, the results of this study provide additional support to the polygenic sex determination hypothesis in sea bass. In addition, identification of sex-ratio QTL may provide new opportunities for sex-ratio control in sea bass.

**Electronic supplementary material:**

The online version of this article (doi:10.1186/s12711-015-0148-y) contains supplementary material, which is available to authorized users.

## Background

Both genetic and environmental factors are involved in the sex determination of various fish species, with sex in some species influenced by both factors [1, 2]. Both XX/XY male heterogametic and WZ/ZZ female heterogametic sex-determining systems exist in fish and, unlike in mammals, individuals with YY and WW genotypes are viable in most fish species tested [3, 4]. The genetic factors that underlie sex determination can range from a single gene to a few sex-determining quantitative trait loci (QTL) or even a complex combination of a large number of genes, as for polygenic traits. Understanding sex determination systems in fish has direct commercial applications, since several commercially important traits, such as growth and age at maturity, are sex-specific in a wide variety of aquaculture fish species.

*Dicentrarchus labrax* (European sea bass) is a highly valuable commercial aquaculture species in Europe, with more than 148 000 t produced in 2014 [5]. The production cycle of *D. labrax* is between 18 and 24 months, with a market size of about 350 to 400 g. Under aquaculture conditions, the percentage of males is usually very high (70 to 90 %), while current evidence suggests that, in wild populations, the sex-ratio is generally balanced [6]. Males tend to grow more slowly than females and lower body weights at harvest time (up to 40 % less) have been reported compared to those of identical cohorts of females [7, 8]. This dimorphism can be interpreted as an advantage to produce large females in a mass-spawning species, as observed in the Atlantic silverside *Menidia menidia* [9].

The *D. labrax* sex determination system depends both on genetic factors and environmental effects, with temperature being considered as the most important environmental effect under hatchery conditions [10, 11]. Studies on induced gynogenesis in *D. labrax* have indicated that the mechanism of sex determination is not explained by a simple monofactorial system with either male or female homogamety [12]. The sex ratio of offspring from masculinised females is not female-biased, which rules out straightforward XX/XY or WZ/ZZ systems [13]. However, Francescon et al. [14] reported that the sex ratio of progeny from meiogynogenetic females is skewed in favour of females.

Environmental temperature influences sex ratio in *D. labrax*, and temperatures above 17 °C during early development (before 60 days post-fertilization) favour the development of males [10]. Higher temperatures (~21 °C), which are typically used during the larval and early juvenile stages in aquaculture hatcheries, are thought to masculinise fish that would have remained as females at lower temperatures. However, unlike what is observed in reptiles, there is no known temperature regime that produces 100 % males or 100 % females in *D. labrax*. In addition, Diaz et al. [15] reported that the growth rate during the stages prior to sex differentiation is linked to the sex ratio, so that faster-growing fish are more likely to be females.

No major sex-determining gene or genetic markers associated with sex have been identified in *D. labrax* as far as we are aware. Strong parental effects, as well as genotype-temperature interactions can modulate the sex ratio in *D. labrax*: the proportion of females resulting from individual crossings may range from 1 to 70 % [10, 16]. Vandeputte et al. [17] provided the first evidence for a polygenic sex-determining system in this species, based on an analysis of between-family variation in sex ratio.

Restriction-site associated DNA (RAD) sequencing is a reduced-representation sequencing platform that exploits high-throughput sequencing methodologies, while the use of barcodes allows multiplexing of samples [18]. This technique can be used for the rapid discovery of thousands of single nucleotide polymorphisms (SNPs) by sequencing parts of the genome at high depth, which offers the possibility to construct high-density linkage maps in a cost-efficient manner. Sex-determining regions have already been identified in *Danio rerio* (zebrafish), *Oreochromis niloticus* (Nile tilapia) and *Hippoglossus hippoglossus* (Atlantic halibut) using RAD-seq [19–22]. In this study, we used RAD sequencing to identify SNPs in F_2_ crosses of *D. labrax* that originated initially from an F_0_ cross between two families with divergent sex ratios. First, a high-density linkage map was constructed, then quantitative trait loci (QTL) mapping and a genome-wide association study (GWAS) were performed. We also carried out a preliminary genomic prediction approach to test the potential of SNP-based selection for increasing either female ratio or body length.

## Methods

### Sample collection and preparation

The fish used in this study originated from an F_2_ cross population of hatchery-reared *D. labrax*. Three F_0_ males and three F_0_ females, which were the offspring of wild West-Mediterranean *D. labrax*, were mated by artificial fertilization in a factorial cross to simultaneously produce eight families (one family was lost), which were reared in common garden conditions; the overall sex ratio in the F_1_ offspring was 43.8 % females. F_1_ males and females were chosen from one family, which was a cross between the F_0_ female that produced the lowest proportion of females (26.6 %) and the F_0_ male that produced the highest proportion of females (58.2 %). Two F_1_ females (Dams 1 and 2) were each crossed to four F_1_ males (Sires 1, 2, 3 and 4) to produce eight families (Table 1). Temperature was kept at 16 °C between 5 and 15 days post-fertilisation (dpf) and at 18 °C between 16 and 42 dpf, then it was increased to 23 to 25 °C between 48 and 98 dpf and finally decreased to an average of 21 °C until the end of the experiment. This protocol was expected to minimise any temperature effect on sex-ratio [11]. Offspring from each female (four paternal half-sib families for each of the two female parents) were reared separately until accurate sexing by visual inspection of the gonads was possible at 16 months of age at which time the fish were sacrificed, weighed, sexed and their body length was measured. A fin sample from each fish was collected and stored in ethanol at room temperature. In total, 175 F_2_ fish (88 males and 87 females) plus the six F_1_ parents and the F_0_ female were used for RAD sequencing (no material from the F_0_ male was available).

**Table 1:** Family summary, descriptive statistics and testing of deviations from equal sex ratio

Dam	Sire	Family	Contribution	Offspring males	Offspring females	Average weight in g (±se)	Average length in mm (±s.e)	*P*-value
Dam 1	Sire 1	1	7.4 %	4	9	194.2 (58.7)	256.5 (20.9)	0.27
Dam 1	Sire 2	2	10.8 %	14	5	179.3 (34.1)	249.5 (15.3)	0.066
Dam 1	Sire 3	3	9.7 %	5	12	181.9 (65.2)	248.6 (31.4)	0.15
Dam 1	Sire 4	4	17.7 %	17	14	178.2 (48.2)	248.7 (19.9)	0.719
Dam 2	Sire 1	5	9.1 %	5	11	148.1 (37)	231.5 (16.1)	0.21
Dam 2	Sire 2	6	12.6 %	14	8	150.2 (54.9)	233.0 (26.8)	0.28
Dam 2	Sire 3	7	10.9 %	3	16	170.0 (67.5)	240.2 (28.3)	0.0059^**^
Dam 2	Sire 4	8	21.7 %	25	13	160.5 (52.8)	239.6 (24.7)	0.074

^**^α = 0.01; se = standard error

### RAD library preparation and sequencing

DNA was extracted from fin samples using the REALPure genomic DNA extraction kit (Durviz S.L.) and treated with RNase. Each sample was quantified by spectrophotometry (Nanodrop), quality assessed by agarose gel electrophoresis, and diluted to a concentration of 50 ng/μL in 5 mmol/L Tris, pH 8.5.

The RAD library was prepared as originally described in Baird et al. [18] and comprehensively detailed in Etter et al. [23], with the minor modifications reported in Houston et al. [24]. Details on the RAD-specific P1 and P2 paired-end adapters and library amplification PCR primer sequences used in this study are in Baxter et al. [25]. Briefly, each sample (0.72 μg parental DNA/0.24 μg offspring DNA) was digested at 37 °C for 40 min with the high fidelity restriction enzyme *Sbf*I that recognises the CCTGCA|GG motif (New England Biolabs; NEB) using 6 U *Sbf*I per μg genomic DNA in 1× Reaction Buffer 4 (NEB) at a final concentration of about 1 μg DNA per 50 μL reaction volume. The samples (12 μL final volume) were then heat-inactivated at 65 °C for 20 min. Individual specific P1 adapters, each with a unique 5 bp barcode (see Additional file 1: Table S1), were ligated to the *Sbf*I digested DNA at 22 °C for 60 min by adding 1.8/0.6 μL (parental/offspring DNA samples respectively) 100 nmol/L P1 adapter, 0.45/0.15 μL 100 mmol/L rATP (Promega), 0.75/0.25 μL 10× Reaction Buffer 2 (NEB), 0.36/0.12 μL T4 ligase (NEB, 2000 U/μL) and reaction volumes made up to 45/15 μL with nuclease-free water for each parental/offspring sample. After heat-inactivation at 65 °C for 20 min, the ligation reactions were slowly cooled to room temperature (over 1 h), then combined in appropriate multiplex pools (see Additional file 1: Table S1). Shearing (Covaris S2 sonication) and initial size selection (250 to 550 bp) by agarose gel separation [24] was followed by gel purification, end repair, dA overhang addition, P2 paired-end adapter ligation and library amplification, exactly as in the original RAD protocol [18, 23]. 150 μL of each amplified library (14 to 16 PCR cycles depending on library) was size-selected (about 350 to 650 bp) by gel electrophoresis [24]. Following a final gel elution step into 20 μL EB buffer (MinElute Gel Purification Kit, Qiagen), 16 libraries (7 animals in the parental library, 12 animals in each of 15 progeny libraries) were sent to the Edinburgh Genomics facility at the University of Edinburgh, UK, for quality control and high-throughput sequencing. Libraries were accurately quantified by qPCR (Kapa Library) and run in four lanes of an Illumina HiSeq 2000, using 100 base paired-end reads (v3 chemistry). Raw reads were processed using RTA 1.12.4.2 (Illumina). Reads were deposited at the EBI Sequence Read Archive (SRA) study ERP004018.

### Genotyping RAD alleles

Reads of low quality (i.e., with a quality score less than 30, while the average quality score was equal to 37), that lacked the restriction site or had ambiguous barcodes were discarded. Retained reads were sorted into loci and genotypes using Stacks software 1.02 described in Catchen et al. [26] The likelihood-based SNP calling algorithm [27] implemented in Stacks evaluates each nucleotide position in every RAD-tag of all individuals and statistically differentiates true SNPs from sequencing errors. Reads were aligned to a draft assembly of the sea bass genome (dicLab v1.0c June 2012) using Bowtie 2 [28] and the generated SAM files were passed to the Stacks wrapper program 'ref_map.pl' to curate RAD loci and call SNPs. Values for the major Stacks parameters were as follows: minimum stack depth (m) = 30; distance between stacks (M) = 2; distance between catalog loci (n) = 1.

### Parentage assignment – general statistics

Parentage assignment was performed with Vitassign V8-5.1 [29] using 200 SNPs and with R/hsphase using all discovered SNPs and allowing for a maximum genotyping error of 3 % [30]. R v.3.0.1 was used for chi-square tests to detect significant deviations from the equal sex ratio and for calculating correlations between phenotypic sex and weight or length. SAS-GENMOD was used to test for sire and dam effects (and their interaction) on sex, using a probit link function and a binomial distribution [31].

### Construction of the linkage map

The linkage map was constructed with Lep-Map [32]. SNPs with a minor allele frequency (MAF) less than 0.05 and SNPs that deviated from the expected Mendelian segregation (*P* < 0.001) were excluded. Linkage groups were formed with a minimum LOD value of 10 using the SeparateChromosomes module of Lep-Map. SNPs within each linkage group were ordered by applying the OrderMarkers module. Map distances were estimated in centiMorgans (cM) using the Kosambi mapping function.

### Heritability estimation

Heritability of phenotypic sex was estimated on the liability scale using R/MCMCglmm [33]. The observed phenotype was considered to follow a binomial distribution. The probit link function was used as follows:

$$ {\mathrm{y}}_{\mathrm{i}}\sim \mathrm{B}\left({\mathrm{probit}}^{\hbox{-} 1}\left({\mathrm{l}}_{\mathrm{i}}\right)\right), $$

where y_i_ is the observed phenotype coded as a binary trait and l_i_ is the latent variable. The latent variable was modelled as follows:

$$ {\mathrm{l}}_{\mathrm{i}}=\mu +{\mathrm{u}}_{\mathrm{i}}+{e}_{\mathrm{i}} $$

where *μ* is the intercept, u_i_ animal random effect that follows ~ N(0,**A**σ_g_^2^), with **A** the pedigree-based relationship matrix and σ_g_^2^ the additive genetic variance, and *e*_*i*_ the residual error. The additive genetic variance was estimated by Markov chain Monte Carlo (MCMC) estimation, using a prior following the *χ*^2^ (1 df) distribution (10 000 000 iterations; 1 000 000 burn-in; 500 thin).

Heritability was estimated using the following formula:

$$ {h}^2=\frac{\sigma_g^2}{\sigma_g^2+{\sigma}_e^2+1}, $$

where *σ*_*g*_^2^ is the previous estimated additive genetic variance and *σ*_*e*_^2^ is the residual variance that was fixed to 1 due to the identifiability issue with binary data [33].

### QTL mapping

Sex-determining QTL were identified by Haley-Knott regression mapping using GridQTL [34, 35]. A half-sib model and a F_2_ sib regression analysis model of the entire pedigree were used, allowing for the existence of one QTL at tested intervals that were 1 cM apart.

The models used had the following general form:

$$ {y}_{ij}={m}_i+{\displaystyle {\sum}_{z=1}^{z=n}{\mu}_z\times {p}_z+{e}_{ij},} $$

where *y*_*ij*_ is the phenotypic sex of individual *j* belonging to full/half sib family i, with males coded as 1 and females as 2, *m*_*i*_ is the F2 – half sib family specific intercept, *μ*_*z*_ is the effect of marker *z*, *n* is the number of possible QTL genotypes of the tested position, *p*_*z*_ is the probability of the inferred genotype (g_z_) based on flanking markers (*p*_*z*_ = Pr(g_z_ |M), M flanking markers) and *e*_*ij*_ is the residual error. Confidence intervals (95 %) were estimated using bootstraps with resampling (10 000 iterations). Two levels of significance were calculated based on chromosome (α = 0.01) or genome-wide thresholds (α = 0.05) by performing 1000 permutations, with the detected QTL referred to as suggestive or significant, respectively [35–37].

### GWAS approach

A GWAS was performed using R/rrBLUP [38] in order to test for SNPs associated with phenotypic sex. The model used was based on Yu et al. [39] and had the following format:

$$ \mathbf{y}=\mathbf{X}\boldsymbol{\upalpha } +\mathbf{Z}\mathbf{u}+\mathbf{e}, $$

where **y** is the vector of the phenotypes, **α** is the vector of marker effects, **u** is the vector of animal random effects ∼ *N*(**0**, **G***σ*_*g*_^2^) and **e** is the vector of residuals. The matrix **G** represents the relationship matrix calculated from SNPs and *σ*_*g*_^2^ the additive genetic variance estimated using REML. **X** and **Z** are incidence matrices relating **y** to **α** and **u**, respectively. The Bonferroni correction was used to correct for multiple testing (Type I error rate = 0.05).

### Prediction of phenotypic sex or length based on estimated breeding values

A preliminary study was conducted to test whether breeding values that were estimated from the additive effects of SNPs, could be used as a predictive measure of phenotypic sex or length, respectively. SNPs with more than 5 % missing data were removed. Missing values of the remaining 4881 SNPs were imputed with R/synbreed [40]. Additive SNP effects were estimated using RRBLUP, BayesA, BayesB [41], BayesC [42] or Bayesian Lasso [43] using R/BGLR [44]. Pedigree-based BLUP [45] was applied using the same software.

The general form of the fitted models was the following:

$$ \mathbf{y}=\boldsymbol{\upeta} +\boldsymbol{\upvarepsilon}, $$

where **y** is the vector of phenotypic records, **η** the linear predictor and **ε** the vector of residuals. In the scenario for the prediction of phenotypic sex, the probit link function was used to connect the underlying latent variable with the linear predictor.

The linear predictor **η** in the case of RRBLUP, BayesA, BayesB, BayesC and Bayesian Lasso had the following general form:

$$ \boldsymbol{\upeta} =\mathbf{1}\upmu +{\mathbf{X}}_{\mathbf{1}}{\boldsymbol{\upbeta}}_{\mathbf{1}}+{\mathbf{X}}_{\mathbf{2}}{\boldsymbol{\upbeta}}_{\mathbf{2}}, $$

where μ is the intercept, **X**_**1**_ and ***X***_**2**_ are design matrices relating the phenotypes to the included fixed effects and markers, respectively, **β**_**1**_ is the vector of regression coefficients for the included fixed effects (length or sex and tank, respectively for each study), and **β**_**2**_ is the vector of marker effects with corresponding priors depending on the model used.

The linear predictor **η** in the case of pedigree-based BLUP had the following form:

$$ \boldsymbol{\upeta} =\mathbf{1}\upmu +{\mathbf{X}}_{\mathbf{1}}{\boldsymbol{\upbeta}}_{\mathbf{1}}+\mathbf{Z}\mathbf{u}, $$

where **u** is the animal random effect that follows ∼ *N*(**0**, **V***σ*_*g*_^2^) where **V** is the pedigree-based relationship matrix. **Z** is the incidence matrix relating **y** to **u**.

The parameters of the above models were estimated by MCMC sampling (100 000 iterations; burn-in: 10 000; thin: 100). Convergence of the resulting posterior distributions was assessed both visually and analytically by the Geweke diagnostic using R/boa v1.1.7 [46].

The dataset was randomly split into a training set (150 individuals) and a test set (25 individuals). This was repeated 100 times to record prediction accuracies for each model tested. The prediction of phenotypic sex was tested by the following naïve approach: animals in the test set that were predicted by the model to have a probability of being female greater than 0.5 were considered as females, while the rest were considered as males. The number of correctly assigned individuals for each model tested was recorded. Τ-tests (right-tailed) were performed to evaluate whether the mean number of correctly assigned individuals was significantly larger (α = 0.05) from that expected by chance alone.

## Results

### RAD reads

In total, 1 156 659 542 raw reads (100 bases long) were produced (578 329 771 paired-end reads, EBI-SRA study ERP004018). After removing low-quality sequences (i.e., with a quality score less than 30), ambiguous barcodes, and orphaned paired-end reads, 76.7 % of the raw reads were retained (886 927 866 reads). Then, assembly and grouping of the sequences into the RAD loci for each individual were performed with the Stacks package [26] and 56 696 unique RAD-tags were retrieved (see Additional file 1: Table S1). In order to maximise the number of informative SNPs and minimise the amount of missing or erroneous data, we used the RAD-tags that were retrieved in at least 75 % of the samples in each family, and that carried only one or two SNPs.

### Parentage assignment- general statistics

All 175 progeny (88 females and 87 males) were assigned to a unique parental pair using Vitassign [29], by allowing a maximum of five mismatches in a test panel of 200 SNPs. Resulting parentage assignments were confirmed with R/hsphase [30], allowing for a maximum genotypic error of 3 %. Sire 1 had the smallest number of progeny, while Sire 4 had the largest number, for both dams. A correlation of 0.96 was found between weight and length, while the correlation between phenotypic sex and weight or length was equal to 0.23. Significant deviations from an equal sex ratio within full-sib families were observed only for the parental pair Dam 2 and Sire 3 (Table 1). The sire effect on sex-ratio was highly significant (*P* < 0.001) but the dam effect was not (*P* > 0.50). The sire-dam interaction was not significant (*P* > 0.50), which indicated that the genetic variation in sex-ratio was additive.

### Linkage map

**Fig. 1:**
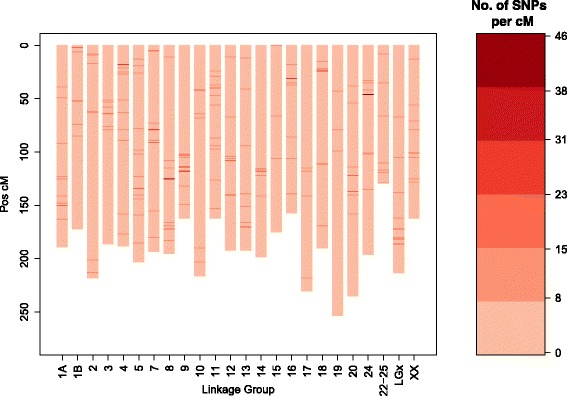
Sea bass linkage map. Heatmap on the right side provides scale of colour coding for the size of SNP clusters

The constructed linkage map consisted of 6706 SNPs that were grouped in 24 linkage groups, in accordance with the number of chromosomes in the *D. labrax* karyotype, with a total length of 4816 cM (Fig. 1; Table 2). Each linkage group corresponded to a different chromosome, as confirmed by comparison with the sea bass genome sequence [47]. In addition, the linkage map included 852 SNPs that were located in unanchored contigs of the sea bass genome (see Additional file 2: Table S2).

**Table 2:** Details of the sea bass linkage map

Corresponding chromosome (seabass_v1.0)	Number of markers	Unique positions	Length (cM)
LG1A	302	125	191.61
LG1B	238	84	163.33
LG2	216	96	204.47
LG3	219	89	174.05
LG4	315	119	191.19
LG5	363	147	206.11
LG6	299	133	243.5
LG7	330	126	245.49
LG8	301	90	156.75
LG9	226	108	259.7
LG10	305	108	218.23
LG11	255	98	192.49
LG12	292	105	181.85
LG13	280	140	188.76
LG14	217	102	216.2
LG15	298	110	173.17
LG16	259	137	227.55
LG17	318	104	199.54
LG18-21	286	147	210.52
LG19	272	111	202.01
LG20	333	113	229.37
LG24	203	76	164.14
LG22-25	321	117	194.33
LGx	258	91	182.57
	6706	2676	4816.93

### Estimation of sex heritability, QTL mapping and GWAS

**Fig. 2:**
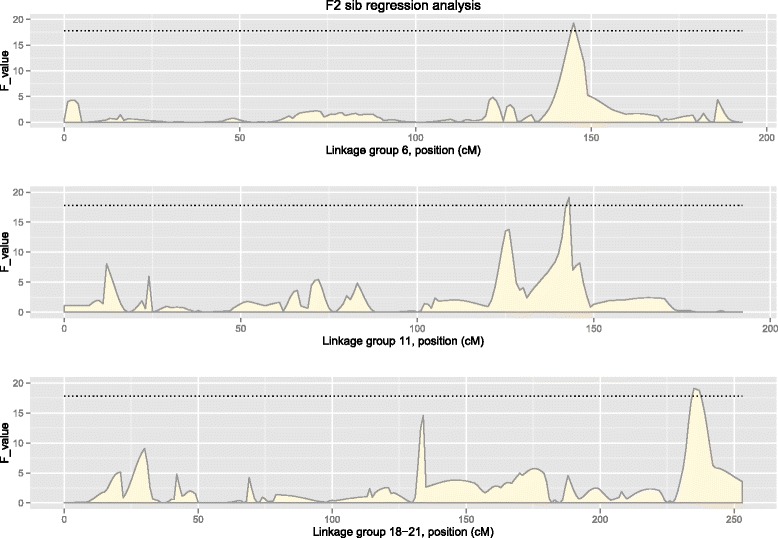
Sex determining QTL (regression analysis). Dotted line corresponds to the genome-wide threshold (α = 0.05; estimated based on 10 000 permutations)

The estimated heritability for phenotypic sex was equal to 0.47 (95 % density interval: 0.15 to 0.79). In the F_2_ sib regression analysis, sex-determining QTL that were significant at the genome-wide level were detected on linkage groups 6, 11 and 18 to 21 (Fig. 2; Table 3), with F values of 19.19, 20.28 and 20.17, respectively. The significance threshold at the genome-wide level had a value of F = 17.8 (10 000 permutations, α = 0.05). Τhe QTL confidence intervals (95 %) spanned regions between 3 and 147 cM in linkage group 6, between 12 and 143 cM in linkage group 11, and between 30 and 237 cM in linkage groups 18 to 21. In addition, one suggested sex-related QTL (significant at the chromosome level; α = 0.01) was detected on linkage group 12 (Table 3).

**Table 3:** Mapped sex-determining QTL using F_2_ sib regression analysis

LG	Position (cM)	LOD	F
6	146	21.28^**^	4.3
11	143	19.19^**^	3.9
12	192	16.19^*^	3.3
18-21	236	20.17^**^	4.1

*Chromosome wide significant (α = 0.01); **Genome-wide significant (α = 0.05)

The maternal half-sib regression model detected suggestive sex-determining QTL in four linkage groups. Two were detected in the first maternal half-sib panel (Dam 1 - linkage groups: 19 and 24; Table 4) and four in the second maternal half-sib panel (Dam 2 - linkage groups: 12, 14, 19 and 24; Table 4). The genome-wide significant threshold F was equal to 19.58 (10 000 permutations, α = 0.05). The paternal half-sib regression model did not detect significant QTL at either the genome-wide level (α = 0.05) or the chromosome level (α = 0.01).

**Table 4:** Mapped sex-determining QTL using half-sib regression analysis

Half-sib family	LG	Position (cM)	F
Dam_1	19	138	16.65^*^
	24	22	16.01^*^
Dam_2	12	186	18.42^*^
	14	121	18.69^*^
	19	109	17.67^*^
	24	90	16.03^*^

*Chromosome wide significant (*P* < 0.01)

**Fig. 3:**
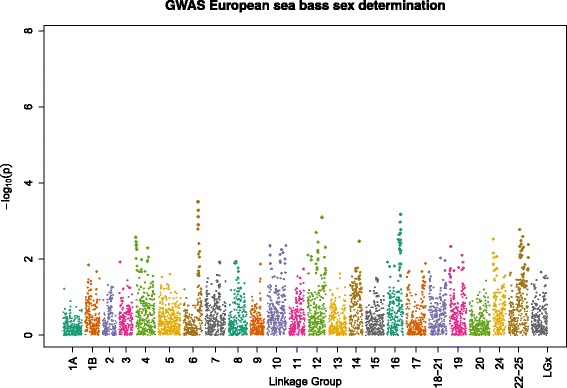
Manhattan plot testing for sex determining regions in European sea bass by GWAS

The GWAS was not able to identify individual SNPs that were significantly associated with phenotypic sex (Bonferroni threshold *P* < 10^−6^). The SNPs with the lowest *p*-values (10^−4^ < *P* < 10^−3^) were located in linkage groups 6 (lowest *p*-value: 10^-3.5^), 12, and 16 (Fig. 3).

### Prediction of phenotypic sex and length based on estimated breeding values

The convergence diagnostics of Geweke and MCMC related graphs did not provide evidence of non-convergence of the estimated parameters (posterior distributions). For prediction of phenotypic sex, all models led to a significantly larger number of correctly assigned animals than expected by chance (Table 5). The application of SNP-based models resulted in a slightly better prediction (mean correct assignment 66 to 67 %) than that achieved using pedigree-based BLUP (mean correct assignment 64 %). For prediction of body length, (Table 6), prediction accuracy was lowest with the pedigree-based BLUP model (0.32) and ranged from 0.41 to 0.44 with the other models.

**Table 5:** Proportions of offspring with sex correctly assigned in the validation sets (25 animals; 100 replicates) and testing of prediction deviations from those expected by chance using t-tests

Model	Assigned correctly (%)	*P*-value
pBLUP	64	<10^−16^
RRBLUP	66	<10^−16^
BayesA	67	<10^−16^
BayesB	67	<10^−16^
BayesC	67	<10^−16^
BayesLasso	67	<10^−16^

**Table 6:** Mean accuracies of predicted length for animals in the validation sets (25 animals; 100 replicates)

Model	Mean accuracy (s.e)
pBLUP	0.32 (0.02)
RRBLUP	0.41 (0.03)
BayesA	0.43 (0.03)
BayesB	0.44 (0.03)
BayesC	0.41 (0.03)
BayesLasso	0.41 (0.03)

## Discussion

*D. labrax* is one of the most important species in Mediterranean aquaculture. The observed sexual dimorphism in growth and the need to control sex-ratio in selective breeding programmes are practical concerns that would benefit from a better understanding of the sex determination system in sea bass. Current evidence, based on the variance of sex-ratio between families, suggests the existence of a polygenic sex determination system [17]. Use of genetic markers offers the potential to directly assess genetic variance and its distribution between putative QTL and a polygenic background.

For *D. labrax*, relatively rich genomic resources are available and a reference genome has recently been publicly released [47]. However, linkage maps that are available for *D. labrax* are mainly based on microsatellites and AFLP (amplification fragment length polymorphisms) and the most recent map consists of 190 microsatellites, 176 AFLP and two SNPs [48, 49]. In this study, we present the first high-density linkage map of *D. labrax* based on 6706 SNPs (2676 unique positions). The number of linkage groups corresponded to the number of chromosomes in the *D. labrax* karyoptype. The accurate grouping of markers was further confirmed by comparison to the reference genome (see Additional file 2: Table S2). The genetic map presented here spans 4816 cM, while the map of Chistiakov et al. [49] has a total length of 1373 cM. We hypothesize that this large increase in size is mainly due to the larger number of markers used. This had already been observed between the map of Chistiakov et al. [49] (368 markers) and the first *D. labrax* linkage map, which was based on 174 microsatellites and spanned 814 cM [48]. The 856 SNPs on our map that are located in unanchored contigs of the current sea bass reference genome (*seabass_V1.0*) should help to improve future versions of the assembly.

A moderate correlation of phenotypic sex with weight and length (r = 0.23) was found. In contrast, Vandeputte et al. [17] reported a relatively strong genetic correlation between weight and sex (r_A_ = 0.5). Successive grading has been shown to produce dominantly female (larger fish) and dominantly male (smaller fish) populations [16, 50], while Diaz et al. [15] showed that a clear relationship exists between growth rate at stages prior to sex differentiation (3 to 4 cm) and sex ratio in sea bass. We estimated a heritability of 0.47 for sex, which is within the range of values reported for sea bass elsewhere, i.e., 0.12 in [51], and 0.62 in [17]. However, different methods were used to estimate heritabilities in these studies, which prevents making meaningful conclusions. It should also be noted that, in our study, the number of parents was small and thus, the estimated heritability cannot be generalized outside the studied pedigree. For comparison, in the study of Vandeputte et al. [17], heritability was estimated using data on 5893 animals from 253 full-sib families.

The F_2_ sib regression analysis detected three genome-wide significant sex-determining QTL on linkage groups 11, 16 and 18. Among these, the best candidate QTL is in linkage group 16, since this region had the highest statistical significance both in the QTL mapping approach and the GWAS. However, since the supporting F-values only just exceeded the estimated genome-wide threshold and since QTL mapping studies tend to overestimate the QTL effect due to the Beavis effect [52], these results should be considered with caution. In addition, the fact that no individual SNP was significant after Βonferroni correction (α = 0.05) requires these QTL to be confirmed in a larger dataset.

Evidence that supports the hypothesis that a polygenic sex determination system exists in *D. labrax* was obtained by testing the efficiency of predicting phenotypic sex based on estimated breeding values. The genomic-based model gave only slightly better predictions than the pedigree-based BLUP. This is most probably due to a combination of factors; primarily the analysis of a relatively small dataset and the response variable being a binary trait (requiring the fitting of generalized linear mixed models). The latter raises additional issues, e.g., the residual variance has to be fixed in order to achieve identifiable estimated parameters. In comparison, when the same models were tested for the prediction of a continuous trait (total body length), the genomic models were clearly more efficient than pedigree-based BLUP. Another issue is that some QTL may be homozygous in some of the parents and thus remain undetected. The fact that paternal half-sib regression does not detect any QTL while most of the variation in sex-ratio is between sire half-sib families would advocate this. In this case, homozygous QTL may contribute to variation between sires, but remain undetected since they do not segregate and thus do not contribute to genomic prediction. Finally, the fact that all genomic models gave similar prediction accuracies, a phenomenon often observed in the study of polygenic traits [41, 53–55], would indirectly support the fact that, in *D. labrax*, sex is a polygenic trait, as hypothesised previously [17].

Although firm conclusions cannot be drawn from this study, the fact that all tested models gave significantly better predictions than that expected by chance alone indicates that further improvement could be possible by increasing genotyping efforts.

## Conclusions

This study presents the first high-density linkage map for the European sea bass. Based on the large number of SNPs (856) that are located in unanchored contigs of the recently published reference genome and thus, that could be positioned, this map will help to improve the existing genome assembly. Overall, the study supports the polygenic hypothesis of sex determination in sea bass of Vandeputte et al. [17]. The families used in this study originated from the West Mediterranean region where significant differentiation exists between populations of *D. labrax*. It is likely that if the unstable nature of the polygenic determinism of sex in *D. labrax* evolves to QTL with larger effects, as suggested by theory [56, 57], this evolution could differ between populations. Searching for population-specific QTL using the same technique as that in this study is, therefore, the next logical step to help unravel the complex genetic sex determination system of this species. Finally, the preliminary genomic prediction results indicate that selection for increased female sex ratios and sizes should be possible.

## Additional files

Additional file 1: Table S1. Origin of samples and barcodes. This file provides details for each sample used i.e., sample ID, family, gender, barcode used, number of raw reads paired-ended and number of RAD-tags. (CSV 23 kb) Additional file 2: Table S2. Detailed positions of individual SNPs in the constructed genetic map. (CSV 281 kb)
